# Probing altered enzyme activity in the biochemical characterization of cancer

**DOI:** 10.1042/BSR20212002

**Published:** 2022-02-04

**Authors:** Mowaffaq Adam Ahmed Adam, Christal D. Sohl

**Affiliations:** Department of Chemistry and Biochemistry, San Diego State University, San Diego, CA 92182, U.S.A.

**Keywords:** cancer, enzyme activity, enzyme kinetics, enzymology, molecular mechanisms

## Abstract

Enzymes have evolved to catalyze their precise reactions at the necessary rates, locations, and time to facilitate our development, to respond to a variety of insults and challenges, and to maintain a healthy, balanced state. Enzymes achieve this extraordinary feat through their unique kinetic parameters, myriad regulatory strategies, and their sensitivity to their surroundings, including substrate concentration and pH. The Cancer Genome Atlas (TCGA) highlights the extraordinary number of ways in which the finely tuned activities of enzymes can be disrupted, contributing to cancer development and progression often due to somatic and/or inherited genetic alterations. Rather than being limited to the domain of enzymologists, kinetic constants such as *k*_cat_, *K*_m_, and *k*_cat_/*K*_m_ are highly informative parameters that can impact a cancer patient in tangible ways—these parameters can be used to sort tumor driver mutations from passenger mutations, to establish the pathways that cancer cells rely on to drive patients’ tumors, to evaluate the selectivity and efficacy of anti-cancer drugs, to identify mechanisms of resistance to treatment, and more. In this review, we will discuss how changes in enzyme activity, primarily through somatic mutation, can lead to altered kinetic parameters, new activities, or changes in conformation and oligomerization. We will also address how changes in the tumor microenvironment can affect enzymatic activity, and briefly describe how enzymology, when combined with additional powerful tools, and can provide us with tremendous insight into the chemical and molecular mechanisms of cancer.

## Introduction

**Figure 1:**
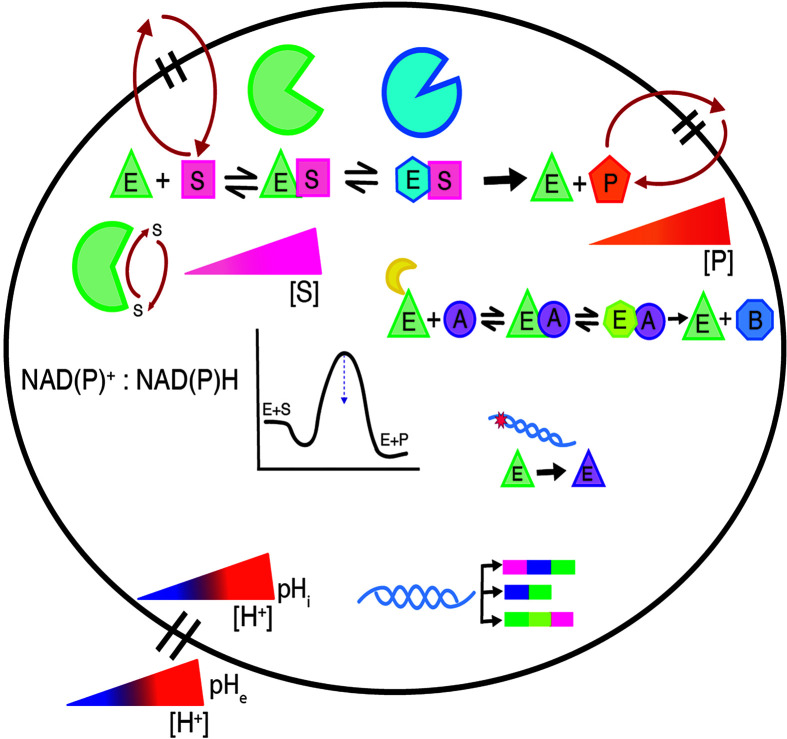
The molecular mechanisms of enzymes in driving cancer A variety of mechanisms can alter or regulate the catalytic activity of enzymes. An example mechanism of enzyme (E, green triangle) binding to substrate (S, pink square), undergoing a conformational change (blue hexagon in the scheme) such as the enzyme substrate complex going from an open (green) to closed conformation (blue) as shown, and finally product (P, orange pentagon) formation and release is shown. Many enzymes have additional, or moonlighting functions (formation of product B, blue octagon, in the moonlighting/non-canonical scheme). In enzymes that have multiple active sites, like the polymerase and exonuclease active sites of many polymerases, changes in substrate partitioning can occur to affect activity (enzyme shown in green with S partitioning between two sites as indicated by red arrows). The activity of enzymes can also be altered at the genetic or transcriptional levels, like the acquisition of somatic mutations or naturally occurring population variants, as indicated by the DNA helix containing a star that results in ethe xpression of a mutant enzyme (E, purple triangle) instead of WT (green triangle), or the use of alternative isoforms, often tissue-specific, as indicated by the three unique transcripts resulting from the DNA helix. Changes in the local cellular environment can also affect enzyme activity, like altered oxidative/reductive potential (shown as changes in NAD(P)^+^:NAD(P)H ratios), changes in substrate or product concentrations (shown as increasing gradients of [S] and [P] that can occur through increased or decreased enzyme activity or transport of these molecules outside the cell via red arrows). Alterations in pH_i_ or pH_e_ (represented as varying concentrations of protons inside or outside the cell), which can occur during the development and progression of cancer, can have important regulatory consequences on pH-sensing enzymes. Establishing the consequences of the changes described in this figure by measuring steady-state kinetic parameters (*k*_cat_, *K*_m_, and *k*_cat_/*K*_m_) is extremely valuable in establishing the role of enzymes in health and disease.

Over 100 years ago, Leonor Michaelis and Maud Menten published their seminal work on the enzymatic properties of invertase [1], ultimately providing us with the useful parameters of assessing and comparing enzymes that we still rely upon today: *k*_cat_, the first-order rate constant for the overall rate-limiting step in turnover; *K*_m_, the concentration of substrate that is required to reach half the maximal rate; and *k*_cat_/*K*_m_, a second-order rate constant describing the catalytic efficiency of enzyme binding to substrate and subsequent turnover to product. These and other kinetic parameters have proved extremely valuable in understanding the role of enzymes in health and disease [2], as they allow us to make many critical comparisons in activity—wildtype (WT) versus mutant, vehicle versus inhibitor treatment, normal versus altered pH, isoform comparisons, variation in substrate or product concentration, etc. As decreased costs and increased accessibility of genetic sequencing facilitated valuable tumor sequencing repositories like The Cancer Genome Atlas (TCGA) [3], we can see the diverse ways in which enzymes can drive tumor formation, tumor growth, and metastasis, as oncoproteins or tumor suppressors, is extraordinary. In this context, these kinetic parameters can be useful to critically analyze the enzyme mutational variants reported in tumors—is this a driver or passenger mutation? How do these mutations affect substrate binding and specificity, and rates of chemistry? What are the consequences in the three-dimensional structure of the enzyme, and how does this affect its kinetic parameters, oligomerization, and/or regulation? How does an ever-changing cellular environment, such as altered substrate concentration or changes in pH, affect enzyme activity? How selective and efficacious are targeted anti-cancer therapies? When viewed via this lens (Figure 1), the utility of these kinetic parameters in understanding some of the fundamental biochemical mechanisms of cancer becomes apparent. Here, we survey examples of enzymes that, through a variety of mechanisms, experience a change in their activity that ultimately leads to a pro-tumor environment (Tables 1 and 2). We focus on enzymes as drivers of cancer rather than the strategies of their therapeutic targeting, which have been reviewed extensively (for example, see [4–10]). Finally, we briefly mention how enzyme kinetics studies are being leveraged to investigate reactions *in situ* and might be employed to understand and predict treatment strategies and prognosis.

**Table 1:** The molecular mechanisms of enzymes involved in DNA binding, synthesis, and repair highlighted in this review

Enzyme	Function	Example(s) of relevant mechanisms that may affect tumor growth or formation	Examples of possible tumor-driving alteration [12]	References
APE1	DNA damage repair: base excision repair, especially in oxidative stress	Changes in kinetic parameters (altered DNA excision rates and substrate-binding affinity) due to mutation	**R237C**, gene amplification	[29,30]
DNMT3A	DNA methyltransferase	Changes in kinetic parameters, oligomerization status due to mutation (leading to decreased tetramer formation, decreased activity, processivity)	**R882H**, R882C, R882P	[79–83]
MGMT	DNA damage repair	Changes in kinetic parameters due to mutation (decreased substrate-binding affinity and changes in methylating reagent sensitivity)	**G132R, G156C**, gene deletion	[37]
REV1	DNA damage repair	Changes in kinetic parameters due to mutation (altered ability to bypass mutations)	**N373S**	[32]
Pol δ	Replicative DNA polymerase	Changes in kinetic parameters due to mutation (altered fidelity and rates of nucleotide incorporation/excision)	**R689W**	[59]
Pol ε	Replicative DNA polymerase	Changes in kinetic parameters due to mutation (altered fidelity and rates of nucleotide incorporation/excision)	**P286R**	[51,55]
Pol ι	DNA damage repair	Changes in kinetic parameters due to mutation (increased or decreased rates of incorporation, altered substrate affinity)	**R96G, I261M, E276K, Y374N**	[47]

Potentially physiologically relevant somatic alterations as reported in [12] such as gene amplification, deletion, or mutation are indicated, and include mutations (in bold), if applicable, highlighted in this review.

**Table 2:** The molecular mechanisms of enzymes involved in signaling and metabolism highlighted in this review

Enzyme	Function	Example(s) of relevant mechanisms that may affect tumor growth or formation	Examples of possible tumor-driving alteration [12]	References
EGFR	Kinase	Changes in kinetic parameters due to mutation via stabilization of active conformation, increased sensitivity to ROS	**L858R**, gene amplification	[19,20,121–123]
GLUD1	Metabolic enzyme	Sensitivity to pH changes leading to altered rates of activity	Gene deletion	[112,113]
IDH1	Metabolic enzyme	Neomorphic activity due to mutation, sensitivity to pH changes	**R132H, R132C**	[66]
LDH	Metabolic enzyme	Moonlighting activity, overexpression/increased activity, sensitivity to pH changes to lead to production of L2HG	Gene amplification	[65,138]
MDH	Metabolic enzyme	Moonlighting activity, sensitivity to pH changes to lead to production of L2HG	Gene amplification	[65]
NQO1	Quinone oxidoreductase	Changes in dynamics due to mutation	**P187S**	[74,76]
PKM2	Kinase	Changes in kinetic parameters due to mutation resulting in altered allosteric regulation, change in sensitivity to ROS	**P119L** **R246S** **G415R**	[147,27,124]

Potentially physiologically relevant somatic alterations as reported in [12] such as gene amplification, deletion, or mutation are indicated, and include mutations (in bold), if applicable, highlighted in this review.

## Role of altered activity in cancer

### Alteration of kinetic parameters in mutational variants

The acquisition of germline line mutations, or, more commonly, somatic mutations, may have a tumor driving role, where the mutation helps provide a pro-tumor environment, or passenger role, where the modification does not play any role in tumor development or growth. The use of kinetic methods is an excellent way to help predict if the resulting mutated enzymes are likely to play a role in cancer. While it is easy to imagine ways in which a point mutation might decrease or ablate enzymatic function, there are also examples of activating mutations. These activating variants are commonly found in regulatory or inhibitory domains within the protein, effectively lifting the brakes on enzymatic activity. For example, many kinases and phosphatases are well-established proto-oncogenes or tumor suppressors. Epidermal growth factor receptor (EGFR) is a transmembrane receptor tyrosine kinase (RTK) belonging to the Erb family, which includes Her2. Deletions and point mutations in EGFR have been well established to drive cancers such as non-small cell lung cancer, and these oncogenic forms are important targets for tyrosine kinase inhibitors (TKIs) [11]. By far, the most common kinase domain mutation is L858R [12], which is found in the activation loop in EGFR and leads to receptor activation and consequently activation of downstream signaling cascades associated with cell proliferation and other pro-tumor pathways [13]. Many elegant structural and kinetic studies have identified the mechanism of activation of WT EGFR and important oncogenic mutational variants, though here we will focus on L858R EGFR. Expression of L858R EGFR is well-established to be sufficient for transforming a variety of cell lines [14–17]. Upon activation, the L858 residue in EGFR experiences a conformational change where it transitions from within a hydrophobic pocket to the surface of the enzyme [18,19]. Crystal structures suggest that somatic acquisition of an arginine at this residue drives the equilibrium to increase the stability of the active conformation [18,19]. Unsurprisingly, these structural changes lead to alterations in the kinetic parameters of L858R EGFR phosphorylation. While there was a ∼4.5-fold increase in *K*_m,ATP_ when comparing WT and L858R EGFR, the *K*_m,peptide_ showed an approximately two-fold decrease [19]. However, overall the catalytic efficiency (*k*_cat_/*K*_m_) of L858R EGFR was increased significantly over WT EGFR, driven primarily by an 18–51-fold increase in *k*_cat_ for the L858R mutant, depending on the peptide substrate used [19]. The EGFR autophosphorylation rate was also affected; rates of autophosphorylation of individual tyrosine residues increased 4-fold to >65-fold in L858R EGFR [20], and there is evidence that changes in phosphorylation pattern may also contribute to the ability of L858R EGFR to transform mammalian cells [21].

Mechanistic studies have also been employed in an attempt to address the role of pyruvate kinase M2 (PKM2) in cancer. PKM2, along with isoforms PKM1, PKL, and PKR, is a glycolytic enzyme that transfers a phosphate group from phosphoenolpyruvate (PEP) to ATP to generate pyruvate. Alternative splicing of the PKM gene leads to two of the isoforms: PKM1, which is a constitutively active tetramer, and PKM2, which can exist as a highly active tetramer or can dissociate to an essentially inactive dimer [22]. As a result of this alternative splicing, PKM2 has additional regulatory features not present in PKM1, including a fructose-1,6-bisphosphate (FBP)-binding pocket that allosterically regulates PKM2’s oligomerization state. Binding of FBP leads to formation of the highly active PKM2 tetramer, while dissociation of FBP and/or protein phosphorylation, acetylation, or oxidation drives PKM2 to its inhibited dimeric form [23]. PKM2 has a complex and likely tumor context-dependent role; this isoform is up-regulated in many cancer types and plays a role in maintaining the metabolic needs of cancer cells [24,25]. Mutations in the PKM gene have also been identified in TCGA, including mutations that affect the regulatory region unique to PKM2 (i.e. absent from PKM1) [26]. Steady-state kinetic experiments have been used to elucidate the mechanistic consequences of these PKM2 mutations. For example, P119L PKM2, which is found in a hinge domain that helps remodel the active site upon substrate binding, showed a five-fold increase in *K*_m,ADP_, leading to decreased catalytic efficiency [27]. R246S PKM2, which is found near the active site, had an increase in *K*_m,PEP_ [27]. G415R is found at the binding surface involved in (active) PKM2 tetramer formation, and this mutation likely affects the allosterically activating role of FBP, as treatment of G415R PKM2 with FBP greatly minimized activation of PKM2 activity [27].

Altered activity in enzymes involved in DNA polymerization and DNA damage repair can often play a role in driving tumor formation and growth. Mutations in apurinic/apyrimidinic endonuclease 1 (APE1) play a role in base excision repair, which is often employed under environments of oxidative stress. APE1 nicks the DNA backbone of AP sites to facilitate base repair by DNA polymerase β (pol β). Mutations in APE1, including R237C, have been implicated in endometrial cancer [28,29]. Alterations in *k*_cat_ are relatively modest (a 2.4-fold decrease in activity relative to WT APE1), though the use of pre-steady-state kinetics identified a 4.4-fold decrease in the rate of DNA cleavage by R237C APE1 compared with WT [30], which is well within the range of activity loss by APE1 to result in a 4–6-fold increase in risk of cancer development [29], and a 2.8-fold increase in affinity for DNA [30]. In some cases, steady-state kinetic methods may not be sufficient to establish the biochemical consequences of tumor-driving enzyme mutations. Steady-state kinetic experiments can have limited utility in polymerases, for example, which often have product release as a rate-limiting step. As a result, altered rates of nucleotide incorporation or excision can often be masked in steady-state kinetic experiments due to slow rates of product release [31].

Similar studies have been undertaken with DNA repair protein REV1 (REV1) mutants [32]. This Y-family polymerase plays a role in translesion synthesis, navigating DNA damage such as abasic sites, G-quadruplexes, and a variety of base adducts [33,34]. In thorough mechanistic work, a dozen germline point mutations implicated in increased cancer risk were assessed for their ability to bypass an assortment of lesions [32]. These mutants were found in a variety of domains, including the fingers, *N*-digit, polymerase-associated domain (PAD), thumb, and insert 1 and insert 2 domains. Interestingly, half of the mutants, which were found in the PAD, thumb, or fingers domain, had up to an eight-fold decrease in catalytic efficiency for dCTP incorporation opposite damaged templates, driven in most cases by a decrease in *k*_cat_ [32]. In contrast, several mutants in the N-digit, insert 1, and insert 2 domains had two- to three-fold increases in catalytic efficiency, primarily through a decrease in *K*_m_ [32]. In addition to DNA lesion bypass and repair, mutations in DNA repair proteins may also affect chemotherapy susceptibility. For example, somatic mutations in O^6^-methylguanine-DNA methyltransferase (MGMT), a protein responsible for removing alkylation at the O-6 position of guanine, have been identified in esophageal and colorectal tumors [35,36]. Two MGMT mutational variants, G132R and G156C, had a 2- and 40-fold decrease in affinity, respectively, for templates containing O^6^-methylguanine, and, as a result, G156C MGMT was far more sensitive to methylating agents [37].

DNA polymerase ι (pol ι) is a Y-family polymerase involved in DNA damage repair via the translesion synthesis pathway. Both decreased and increased activity of this polymerase have been implicated in a variety of cancers [38–43], with both decreased and increased activity likely contributing to a mutator phenotype due to its role in DNA damage repair and relatively low native fidelity, respectively [44–46]. A series of *N*-terminal deletion variants and point mutations in the finger (R96G), thumb (I261M, E276K), and PAD (Y374N) of pol ι, all identified in humans, were kinetically characterized in terms of efficiency of correct and incorrect incorporation opposite a native or damaged DNA template [47]. One deletion mutant, Δ1-25, had increased activity opposite abasic sites, with up to a ten-fold increase in catalytic efficiency (*k*_cat_/*K*_m_) in incorporation opposite a DNA lesion, or a seven-fold increase in DNA substrate affinity. In contrast, R96G pol ι had a notable decrease in catalytic efficiency for incorporation opposite an abasic site, ranging from 5- to 72-fold depending on the type of DNA primer/template substrate used [47]. Only through mechanistic enzymology studies can we elucidate how tumor-relevant mutations in DNA repair enzymes can lead to mutator phenotypes, persistence of DNA damage, or chemotherapy (in)sensitivity, as we can distinguish between increased efficiency of error-prone repair, decreased efficiency of damage repair or translesion synthesis, loss of fidelity, incorporation or repair of chemotherapies, and other important mechanisms.

Cancer-driving mutations are not limited to enzymes involved in DNA repair; replicative polymerase mutants have also been shown to increase genome instability in the development of cancer. Rigorous work in yeast have systematically identified the interesting consequences of DNA polymerase ε (pol ε) and DNA polymerase δ (pol δ) mutations, with many findings reviewed here [48]. Mutations in pol ε and pol δ lead to a hypermutator phenotype, driving many colorectal and endometrial cancers [12,49–54]. Steady-state kinetic analysis in yeast pol ε has unraveled one interesting mystery—how a single point mutation in the exonuclease domain of pol ε leads to a more severe hypermutated phenotype in yeast than full ablation of exonuclease domain activity [51]. While some residual exonuclease activity is retained in the yeast analog of P286R pol ε, an exonuclease domain mutation representing the most common pol ε mutant found in tumors, polymerization is more efficient than that seen in WT pol ε [55]. It was posited that the DNA’s access to the proofreading domain becomes restricted in this mutant, leading to higher polymerase activity and accumulation of genomic mutations [55], though other factors are also at play, including suppression of mismatch repair [56]. This is in a sense a change in the partitioning of substrates in the two active sites. Interestingly, pol ε exonuclease domain mutations are predictive of patient progression-free survival, with better outcomes seen in the presence of these mutations [52]. The human R689W pol δ mutant, a polymerase domain mutant that is an established driver of colorectal cancer [12,57,58], has been explored in yeast [59]. This mutant retains exonuclease activity, but is more likely to incorporate incorrect nucleotides and is more prone to less-extended products as compared with WT pol δ [59].

### Moonlighting and neomorphic activity

Many proteins can display moonlighting activity in which the enzyme has additional functions beyond its more established catalytic role. These new activities do not result from enzyme promiscuity or gene/mRNA alterations like translocations or alternative splicing, and likely arose via evolution [60]. An important metabolite produced primarily through moonlighting activity is 2-hydroxyglutarate (2HG), which can ordinarily be found at very low levels primarily through hydroxyacid-oxoacid transhydrogenase (HOT) activity [61]. This metabolite, in both *L* and *D* forms (L2HG and D2HG), does not have established physiological activity and can potentially serve as a competitive inhibitor of α-ketoglutarate (αKG)-dependent enzymes, which include enzymes involved in DNA and histone demethylation, DNA repair, hypoxia response, and collagen processing [62,63]. Such inhibition can potentially lead to cellular de-differentiation, and 2HG has been posited to be an oncometabolite [61]. Degradation of L-2-hydroxyglutarate (L2HG) and D-2-hydroxyglutarate (D2HG) into αKG is undertaken by stereoisomer-specific 2-dehydrogenases, indicative of the potentially dangerous nature of this metabolite, though in several cancer types accumulation of these metabolites can presumably overwhelm these enzymes [61,64].

Both lactate dehydrogenase A (LDHA), which normally catalyzes the conversion of pyruvate into lactate, and malate dehydrogenase (MDH), which typically interconverts oxaloacetate and malate, can catalyze the reduction of αKG to L2HG [65]. In the case of LDH, this activity is heightened in acidic pH (see below) due to pushing the equilibrium towards a protonated carboxylate form of αKG, which is posited to facilitate active site binding due to a stronger hydrogen bond with Q100 in LDH and decrease in *K*_m_ [65], rather than changes in amino acid protonation states. The most striking source of 2HG, specifically D2HG, is from isocitrate dehydrogenase 1 and 2 (IDH1, IDH2) mutations [66]. These mutants represent a class of tumor drivers that facilitate cancer development and growth through the neomorphic conversion of αKG into D2HG, leading to de-differentiation and DNA and histone hypermethylation [61,66]. These mutations most commonly affect residue R132 in the case of IDH1, and R140 and R172 in IDH2, leading to loss of the normal conversion of isocitrate into αKG and gain of the neomorphic activity [66–68]. In IDH1, R132 is responsible for coordinating the C3-carboxylate of isocitrate, the unique feature when comparing isocitrate (the canonical reaction substrate) and αKG (the neomorphic reaction substrate) [69]. Interestingly, the frequency of IDH1 mutations present in patients appears inversely related to D2HG levels quantified in glioma tissue, with R132G IDH1 mutations leading to the highest concentrations of D2HG in tumors, followed by R132C and R132H, and mutation frequency trends of R132H > R132C > R132G IDH1 in glioma patients [12,70]. We recently showed that these kinetic features, at least in part, help drive these D2HG concentration variations seen in patients, with catalytic efficiency (*k*_cat_/*K*_m_) of physiologically relevant IDH1 mutants, from most efficient to least efficient, showing the following trend: R132Q > R132L >> R132V > R132S > R132G > R132C > R132H [67,68]. These changes in catalytic efficiency, driven primarily by changes in *K*_m_, represent an interesting example of tuning catalytic efficiency by mutational variants affecting a single residue, and highlight the intriguing possibility of tying kinetics to patient prognosis (see below).

### Protein conformations and dynamics

Structural features of enzymes dictate their kinetic parameters, and this can extend beyond the proper positioning of active site residues for optimum substrate binding and catalysis. Protein dynamics can encompass the transition between physiologically relevant conformational states, oligomerization, and allosteric regulation, all of which can affect kinetic parameters. There are several interesting examples of tumor-driving mutations in enzymes that disrupt protein mobility and/or oligomerization, ultimately translating into measurable changes in kinetic parameters that affect protein kinetics in physiologically relevant ways. NAD(P)H quinone oxidoreductase (NQO1) is a cytosolic dimeric enzyme that performs two-electron reductions of molecules like quinones using nicotinamide adenine dinucleotide (reduced) (NADH) or nicotinamide adenine dinucleotide phosphate (reduced) (NADPH), and flavin adenine dinucleotide (FAD) [71]. This protein can also bind to tumor-suppressing proteins like p53, resulting in their stabilization due to protection against proteasomal degradation [72]. NQO1 amplifications, mutations, and deletions have been identified in a variety of cancers [12], and P187S NQO1 in particular has been associated with an increased cancer risk [73] and leads to decreased cellular NQO1 activity and protein concentration. The mutational variant 187S NQO1, located in the *N*-terminal catalytic domain, does not affect a residue that directly interacts with substrates or cofactors. However, this mutant showed a ∼400-fold decrease in affinity for FAD, and ∼200-fold decrease in *k*_cat_ [74]. Proteolysis experiments suggest that P187S NQO1 experienced major changes in mobility in the absence of bound FAD or a stabilizing quinone inhibitor, dicoumarol, as shown by increased sensitivity to thermolysin treatment and unique cleavage patterns [74]. Further, this mutant had decreased thermal stability that could be rescued by FAD [75]. Molecular dynamics (MD) simulations suggested that P187S NQO1 had increased flexibility in the FAD and NAD(P)H binding pockets and at the dimer interface, likely explaining the severe decrease in the enzyme’s activity and affinity for its cofactor [74,76]. Rescue of stability by adding FAD or stabilizing inhibitors was confounded by the enzyme’s very low affinity for these compounds, and cell culture assays suggested these changes in mobility led to an increase in the proteasomal degradation of NQO1 [74,76]. Together these structure/function studies elucidated varied and complex mechanisms associated with this loss-of-function mutational variant.

DNA methylation is a critical mechanism of epigenetic transcriptional regulation. DNA methyltransferase 3A (DNMT3A) is responsible for the *de novo* methylation at C5 of cytosine (5mC) in CpG regions, and somatic mutations of DNMT3A have been implicated in >20% of cases of acute myeloid leukemia (AML) [12,77,78]. Systematic biochemical analysis of the myriad DNMT3A mutants identified in patients revealed a variety of cancer-driving mechanisms that were not always readily predicted using structural assessment [79–83]. Many DNMT3A mutants led to an increase or decrease in *k*_cat_ of CpG and non-CpG methylation [81]. However, some mutants affected DNMT3A oligomerization. DNMT3A is thought to be catalytically active as a homotetramer, and several tumor-relevant mutations are found at these protein–protein interface sites. R882H DNMT3A, the most common point mutation found in mutant DNMT3A-driven AMLs, prevents tetramer formation, leading to an 80% loss in methyltransferase activity [82,83]. This also appears to affect the processivity of CpG methylation [79] and, potentially, binding of regulatory proteins like DNMT3L and p53 [80].

## Role of the cellular environment in modulating enzyme activity in cancer

### pH as a regulator of enzyme activity

In addition to tumor-driving somatic and germline mutations, environmental changes in the cell, like altered pH, can arise during the development and progression of cancer. Protein protonation is a unique post-translational modification (PTM) in that it only requires an appropriate change in local pH and is rapidly reversible, in contrast with PTMs that require an enzymatic reaction and/or energy input [84]. Changes in pH can be suitable to regulate protein activity; for example, changes in intracellular pH (pH_i_) can alter the protonation state of ionizable residues in enzymes, potentially leading to changes in structural conformation, protein–ligand or protein–protein interactions, protein stability and/or activity [82,84]. Many normal and disease-relevant cellular processes are associated with a change in pH, such as apoptosis, immune response, oxidative stress, differentiation, migration, as well as tumor growth, progression, and metastasis [85–96]. This pH sensitivity can often be driven by the presence of buried ionizable residues such as arginine, lysine, histidine, aspartic acid, or glutamic acid if the residue’s p*K*_a_ value is in a physiologically relevant range, allowing it to sense small changes in pH (for cancer cells, usually in the range of pH 6.7–7.6) [97–99]. Proteins that detect and respond to changes in pH by altering activity are known as pH sensors [100], with examples including members of ATPases, GTPases, kinases, and metabolic enzyme families [101–105].

Many pH sensors are found in the mitochondria, an organelle that experiences a variety of important physiological pH changes as a result of signaling, metabolism, and other processes. For example, the mitochondrial enzyme glutamate dehydrogenase 1 (GLUD1) catalyzes the reversible NAD(P)^+^-dependent oxidative deamination of glutamate to form αKG and ammonium ion [106]. GLUD1 plays a pro-survival role in cancers such as gliomas by driving anaplerosis, lipid biosynthesis, cell proliferation, and metastasis [107,108], with overexpression of GLUD1 and GLUD2 being particularly important in mutant IDH1-driven cancers to compensate production of αKG required for energy and metabolite production and lipid biosynthesis [109]. GLUD1 activation can also support cancer cell growth through NH^+^_4_ fixation, as increased concentrations of NH_4_^+^ can support proliferation, migration, and survival of metastatic cancer cells [110,111]. Regulation of GLUD1 is complex; high concentrations of substrates can inhibit activity by forming abortive complexes in which NAD(P)H binds in the active site at more alkaline pH to inhibit oxidative deamination, or NAD(P)^+^ binds at lower pH to inhibit the reductive amination [112]. This regulation is further tuned by binding of ADP to an allosteric pocket in GLUD1, which can also be activating or inhibiting depending on pH and substrate concentration [112]. At pH levels of 7.0 and higher, activation of oxidative deamination is seen at lower ADP concentration by destabilizing these abortive complexes [112]. At pH levels below 7.0, ADP binding is associated with inhibition of oxidative deamination, though in the absence of ADP, oxidative deamination by GLUD1 increases upon increasing pH [112]. Further, decreasing the pH also increases the *K*_m_ for ammonia, inhibiting the reductive amination reaction [113].

**Figure 2:**
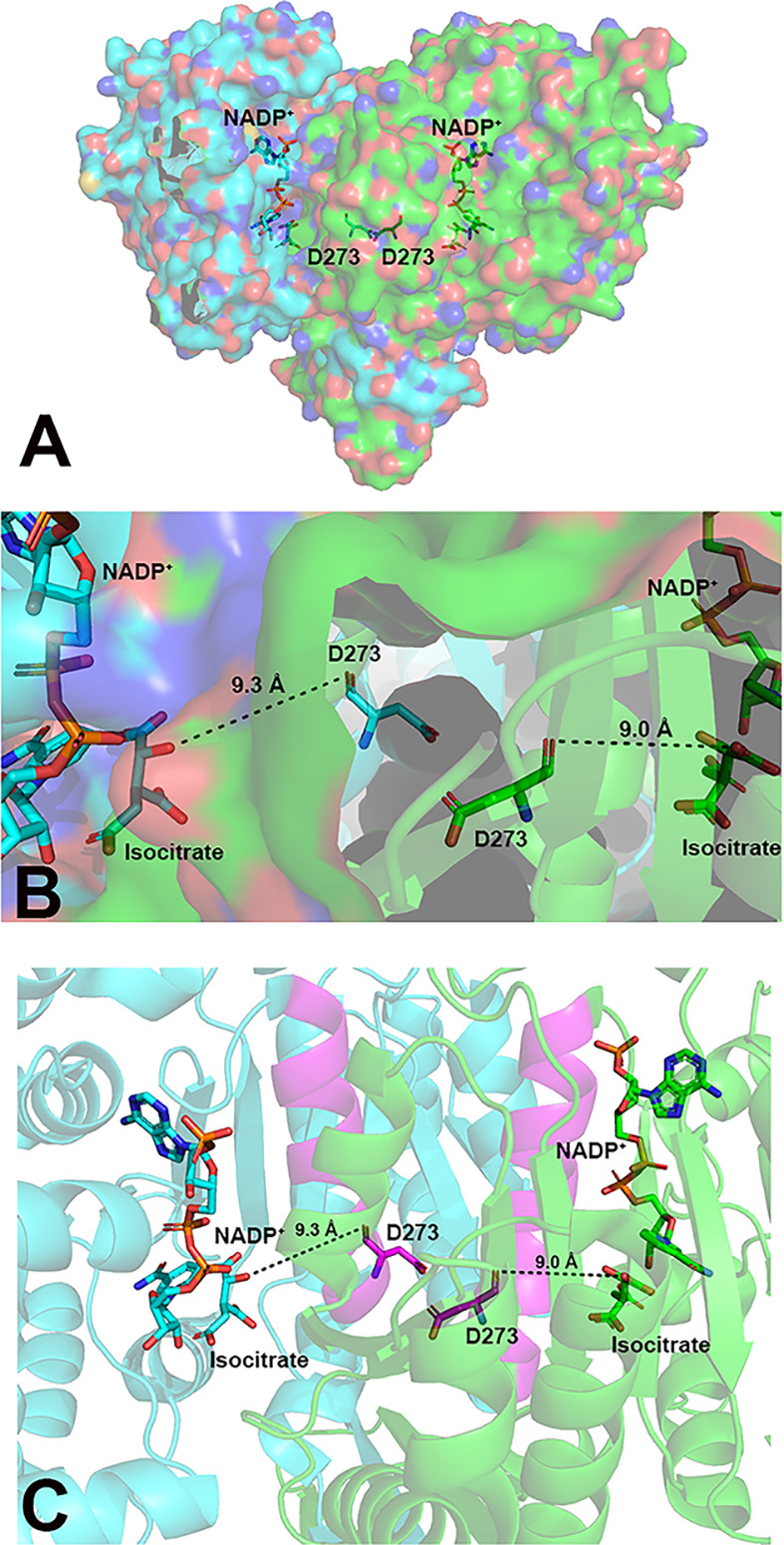
pH sensing in IDH1 (**A**) IDH1 is a homodimer (monomers are shown in cyan and green), with each monomer containing an active site where substrates NADP^+^ and isocitrate bind (shown in sticks). (**B**) A zoomed-in view of IDH1 highlighting the proposed pH-sensing residue D273 in each monomer, which is found near the dimer interface [102]. The shortest distances from D273 to substrate are shown, indicating that this residue is too far to directly interact with the substrates. (**C**) D273 is located in the α10 regulatory domain, highlighted in magenta. This domain plays an important role in remodeling the active site to a catalytically competent or inhibitory conformation [69]. This figure was generated from PDB 1T0L [69] using Pymol [146].

IDH1 catalyzes the reversible oxidative decarboxylation of isocitrate to αKG while reducing NADP^+^ to NADPH, which can support anaplerosis, provide reducing power, and facilitate lipid metabolism [114–116]. Recently, we described a mechanism of pH regulation of WT IDH1 catalysis, a long-described phenomenon whose mechanism was not well understood. We found that the catalytic rate of the forward reaction (isocitrate to αKG) was increased upon increasing pH [102]. To establish the mechanism of pH sensitivity, we identified a buried aspartic acid residue in WT IDH1, D273, that sensed local changes in pH likely by undergoing a change in protonation state (Figure 2) [102]. This residue is found in the α10 regulatory domain, which undergoes a conformational change to restructure the active site from an inhibitory to a catalytically competent form (Figure 2) [69]. When D273 was mutated to a non-ionizable form, there was a significant decrease in the catalytic efficiency of IDH1 and, more importantly, catalysis was no longer dependent upon pH. As described previously, mutant IDH1 is also an established driver, with mutations affecting R132 resulting in the ablation of the native IDH1 activity of isocitrate and α-ketoglutarate (αKG) interconversion, and facilitating a neomorphic reaction: the reduction of αKG to D2HG, an oncometabolite [66]. This reaction has also been recently posited to be pH-sensitive by Sesanto et al. [117]. Interestingly, this pH regulatory mechanism appears to be modulated by monomer–monomer interactions; both mutant and WT IDH1 catalysis require that the enzyme is in its active dimeric form (Figure 2), and generation of mutant/WT heterodimers (WT/R132H IDH1 heterodimers) was shown to be pH-sensitive, while R132H/R132H homodimer formation appeared to be pH-insensitive [117].

### Oxidative stress as a regulator of enzymatic activity

The rapid proliferation of cells observed in tumor growth can lead to hypoxia and increased reactive oxygen species (ROS) production, and cells often adapt to these stresses by launching angiogenesis pathways and hypoxia signaling networks [118]. Increased ROS are a common feature of cancers with their concentrations likely tuned by tumors; on one hand ROS can serve as second messengers to regulate pathways involved in cell growth, survival, metabolism, inflammation, etc., but tumors also typically evolve strategies to detoxify high levels of ROS, suggesting a finely held balance is required for tumor growth and progression [119]. Oxidative stress can affect the activity of proteins in a variety of ways, including PTMs. While broad discussion of the role of PTMs in altering catalytic activity is beyond the scope of this review, we will highlight a few ROS-driven PTMs that alter the activity of tumor drivers. For example, EGFR can be reversibly *S*-sulfenylated (Cys-SOH) at C797, a residue of tremendous therapeutic interest [120], by the second messenger H_2_O_2_ [121,122]. As a result, EGFR becomes activated by H_2_O_2_ through a dose-dependent increase in *k*_obs_ [121–123]. Interestingly, MD simulations predicted that this appears to be driven by conformational changes in the catalytic loop of EGFR; *S*-sulfenylation was predicted to facilitate a new electrostatic interaction with R841 in EGFR, presumably promoting catalysis [123]. Cysteine oxidation is also a regulatory strategy for PKM2; oxidation of C358 by ROS causes the reversible inhibition of PKM2 [124]. Interestingly, oxidation of C358 is thought to destabilize the homotetrameric form needed for full catalytic activation (see above) [124]. As a result of this PKM2 inhibition, an increase in glucose flux into the pentose phosphate pathway can drive production of NADPH that is needed to regenerate oxidized glutathione to increase antioxidant pools [124].

## Enzyme activity in treatment and diagnosis

### Evaluating efficacy and toxicity of asparaginase treatment in acute lymphoblastic leukemia

For decades, asparaginase treatment has been a critical component of the effective therapeutic strategies for acute lymphoblastic leukemia (ALL), which is generally fully curable in pediatric patients [125]. In ALL, cancer cells become addicted to asparagine for protein synthesis, and thus patients are treated with recombinantly expressed asparaginase that hydrolyzes asparagine to aspartic acid and ammonia. Asparaginase can also hydrolyze glutamine, and it has been thought that this contributes to some of this therapeutic’s toxicity [126]. A series of asparaginase mutants were designed to minimize glutaminase activity while preserving asparaginase activity. The A31I/E63Q/S254Q triple mutant affectively tuned this selectivity, with only a 1.6-fold decrease in catalytic efficiency for asparaginase activity, and an impressive ∼1900-fold decrease in catalytic efficiency for glutaminase activity [127]. Importantly, asparaginase mutants maintained efficacy in mice with ALL, and toxicity was shown to be decreased [128]. However, there are several *in vitro* and *in vivo* studies suggesting that while glutaminase activity is likely behind much of the observed cytotoxicity, it may nonetheless still play some role in efficacy [129,130], and more complex models are likely needed to unravel the role of these two activities in patients.

### New tools and ideas in connecting kinetics to disease

Combined structural and computational approaches have also been employed to predict the effects of mutations identified in tumors as a higher throughput method of sorting passenger from driver mutations. As cancer-driving mutations are more likely to be found on protein surfaces and binding interfaces versus the protein core [131,132], a study was undertaken to map 695 point mutations from 598 genes reported in glioblastomas onto an interactome map of established protein–protein, protein–nucleic acid, and protein–ion binding interfaces, and the effects on complex stability and binding energy were modeled and calculated [133]. This study showed that these missense mutations primarily had a destabilizing effect on protein–protein interactions, driven mostly by less favorable electrostatics. Interestingly, mutations on binding interfaces tended to have higher physicochemical distances and often affected arginine with frequent mutation to cysteine [133]. These calculations were also useful in predicting oligomer stability. Heterozygous IDH1 mutations have been shown to have higher activity as WT/mutant heterodimers versus mutant/mutant homodimers [134], and here, modeling indicated that the R132H IDH1 mutation better stabilized the inactive conformation of the mutant/mutant IDH1 than the WT/mutant homodimer [133]. There is also growing evidence that there is a connection between patient prognosis to IDH1 mutation. For example, Tesileanu et al. showed that patients with R132 mutations other than R132H IDH1 had increased DNA methylation and better survival outcomes than patients with R132H IDH1 driven tumors [135]. We have also shown that IDH1 mutational variants vary in binding to selective mutant IDH1 inhibitors *in vitro* [67]. Specifically, we posited that retention of the normal isocitrate to αKG activity by R132Q IDH1 would lead to loss of mutant IDH1 inhibitor binding. Indeed, R132Q IDH1 showed a loss of affinity for all selective mutant IDH1 inhibitors tested, yielding biochemical and cellular IC_50_ values similar to WT IDH1 [67].

As systematic testing of the enzymatic activity of the myriad genetic alterations reported in TCGA is often not practical, there is a great desire to develop computational tools that predict driving versus passenger genetic alterations. For example, MetOncoFit uses metabolic modeling and machine learning to analyze TCGA data to integrate and predict the catalytic and network topological features driving the metabolic reprogramming that drives tumors [136]. Oruganty et al. showed that *k*_cat_ was the best predictor examined for changes in enzyme expression levels in all cancer types, with increased *k*_cat_ correlating with metabolic enzyme gene up-regulation, primarily through increased copy number or increased gene expression, and decreased *k*_cat_ correlating with down-regulation [136]. Interestingly, the *k*_cat_ and differential expression connection was also useful in predicting survival, supportive of the idea of metabolic rewiring giving tumors an adaptive edge [136].

## Future perspectives

Enzyme kinetics technologies have the potential to move even beyond the important roles of classifying driver versus passenger mutations, or identifying the molecular mechanisms of tumor drivers. One exciting area of exploration is the connection between enzymatic mechanisms and patient prognosis and disease severity based on a patient’s particular mutational variant. While focusing on an isolated feature of one tumor driver amid the complex and heterogeneous features of the cancer cell, tumor, and patient is inappropriately simplistic, there is nevertheless some evidence of cases where changes in mechanistic features of enzymes can tune disease severity more like a rheostat than as an on/off switch. For example, amplification of LDHA is commonly observed in tumors [12], and leads to increased lactate dehydrogenase (LDH) levels and activity. Interestingly, increased serum levels of LDH, and thus increased LDH activity, in pancreatic ductal adenocarcinoma (PDAC) patients correlates to worse survival if levels are not decreased prior to chemotherapy [137]. Similarly, there is evidence that ovarian cancers that are higher stage (III, VI versus I, II) and higher grade (G2, G3 versus G1) have higher LDH activity [138]. Beyond amplifications, there is also interest in connecting disease severity to mutation type. For example, polymerases represent an exciting class for this type of work, particularly when combined with pre-steady-state kinetics methods that allow differentiation between the individual steps of an enzyme mechanism that may be affected by the mutation. Indeed, such work has been performed that links mutations in polymerase γ and the severity of mitochondrial disorders [139]. Excitingly, CRISPR-Cas-based models of tumor-relevant Pol ε mutants have facilitated modeling of mutational signatures in human cells, connecting particular mutants to degree of hypermutation [56,140]; such work is ideal to pair with mechanistic enzymology approaches for predicting kinetics/prognosis connections.

A second exciting area of research is applying kinetic techniques to single-cell and tissue imaging technology for *in situ* enzymology that can aid diagnosis and establish molecular mechanisms of disease. For example, in enzyme histochemistry [141], a confocal microscope can be used to measure rates of enzyme reactions by treating intact tissue with a specific substrate and then monitoring cofactor (NAD(P)^+^/NAD(P)H, etc.) oxidation or reduction with a redoxsensitive dye. As a proof of concept of the power and potential of this technology, changes in metabolic enzymes were measured in a comparison between normal and colon tumor tissue [142]. Near-infrared fluorescence (NIRF) molecular imaging has been used to measure endogenous activity of overexpressed NQO1 in tumor models by coupling turnover to near-infrared fluorescent probes, allowing researchers to directly image and detect tumors in mouse models of lung cancer [143]. Proteomics methods are also powerful for probing kinetics *in situ*. For example, liquid chromatography coupled to tandem mass spectrometry (LC-MS/MS) has been used to measure enzymatic activity of specific enzymes within complex metabolic pathways important in cancer like glycolysis [144]. Spatial and kinetic information can be measured by using enzyme activity matrix-assisted laser desorption/ionization imaging mass spectroscopy (EA-MALDI-IMS) to measure enzyme catalysis in tissue sections. In this case, protease and kinase activities were monitored after applying substrates to tissue, and then the mass of substrates and products were measured and mapped to the sub-tissue location [145]. The combination of enzymology with powerful imaging and proteomics technologies is an exciting strategy for understanding enzyme activity within the complex and rapidly changing environments of cells and tissues, and has tremendous potential in diagnostics and tumor imaging to greatly enhance our understanding of cancer.

## Abbreviations

αKG: α-ketoglutarate
2HG: 2-hydroxyglutarate
ALL: acute lymphoblastic leukemia
AML: acute myeloid leukemia
APE1: apurinic/apyrimidinic endonuclease 1
DNMT3A: DNA methyltransferase 3A
D2HG: D-2-hydroxyglutarate
EGFR: epidermal growth factor receptor
FAD: flavin adenine dinucleotide
FBP: fructose-1,6-bisphosphate
GLUD1: glutamate dehydrogenase 1
IDH: isocitrate dehydrogenase
LDH: lactate dehydrogenase
LDHA: lactate dehydrogenase A
L2HG: L-2-hydroxyglutarate
MD: molecular dynamics
MGMT: O^6^-methylguanine-DNA methyltransferase
NADPH: nicotinamide adenine dinucleotide phosphate (reduced)
NQO1: NAD(P)H quinone oxidoreductase
PAD: polymerase-associated domain
PEP: phosphoenolpyruvate
PKM: pyruvate kinase M
Pol β: polymerase β
Pol δ: polymerase δ
Pol ε: polymerase ε
Pol ι: polymerase ι
PTM: post-translational modification
ROS: reactive oxygen species
TCGA: The Cancer Genome Atlas
WT: wildtype
